# Six months vitamin K treatment does not affect systemic arterial calcification or bone mineral density in diabetes mellitus 2

**DOI:** 10.1007/s00394-020-02412-z

**Published:** 2020-10-17

**Authors:** Jonas W. Bartstra, Fieke Draaisma, Sabine R. Zwakenberg, Nikolas Lessmann, Jelmer M. Wolterink, Yvonne T. van der Schouw, Pim A. de Jong, Joline W. J. Beulens

**Affiliations:** 1 Department of Radiology, University Medical Center Utrecht, Utrecht University, Utrecht, The Netherlands; 2 Julius Center for Health Sciences and Primary Care, University Medical Center Utrecht, Utrecht University, Utrecht, The Netherlands; 3 Diagnostic Image Analysis Group, Department of Radiology and Nuclear Medicine, Radboudumc, Nijmegen, The Netherlands; 4 Department of Applied Mathematics, Technical Medical Centre, University of Twente, Enschede, The Netherlands; 5 Department of Epidemiology and Data Science, Amsterdam University Medical Centers, Location VUmc, Amsterdam Public Health and Amsterdam Cardiovascular Sciences Research Institutes, Postbox 7057, 1007 MB Amsterdam, The Netherlands

**Keywords:** Randomized controlled clinical trial, Type 2 diabetes, Cardiovascular disease, Vitamin K, Arterial calcification, Bone mineral density

## Abstract

**Purpose:**

Vitamin K-dependent proteins are involved in (patho)physiological calcification of the vasculature and the bones. Type 2 diabetes mellitus (DM2) is associated with increased arterial calcification and increased fractures. This study investigates the effect of 6 months vitamin K2 supplementation on systemic arterial calcification and bone mineral density (BMD) in DM2 patients with a history of cardiovascular disease (CVD).

**Methods:**

In this pre-specified, post hoc analysis of a double-blind, randomized, controlled clinical trial, patients with DM2 and CVD were randomized to a daily, oral dose of 360 µg vitamin K2 or placebo for 6 months. CT scans were made at baseline and follow-up. Arterial calcification mass was quantified in several large arterial beds and a total arterial calcification mass score was calculated. BMD was assessed in all non-fractured thoracic and lumbar vertebrae.

**Results:**

68 participants were randomized, 35 to vitamin K2 (33 completed follow-up) and 33 to placebo (27 completed follow-up). The vitamin K group had higher arterial calcification mass at baseline [median (IQR): 1694 (812–3584) vs 1182 (235–2445)] for the total arterial calcification mass). Six months vitamin K supplementation did not reduce arterial calcification progression (*β *[95% CI]: − 0.02 [− 0.10; 0.06] for the total arterial calcification mass) or slow BMD decline (*β* [95% CI]: − 2.06 [− 11.26; 7.30] Hounsfield units for all vertebrae) when compared to placebo.

**Conclusion:**

Six months vitamin K supplementation did not halt progression of arterial calcification or decline of BMD in patients with DM2 and CVD. Future clinical trials may want to pre-select patients with very low vitamin K status and longer follow-up time might be warranted. This trial was registered at clinicaltrials.gov as NCT02839044

## Introduction

Vitamin K is a fat-soluble vitamin that is involved in the carboxylation and activation of several vitamin K-dependent proteins. It occurs in two different forms, phylloquinone (vitamin K1) and menaquinone (vitamin K2), which differ in the length and degree of saturation of the side chain [1]. Phylloquinones are mainly derived from green vegetables, whereas menaquinones are mainly derived from animal products like cheese and meat [2]. Phylloquinones and menaquinones both have a methylated napthoquinone ring structure, which is the functional group, but menaquinones have a longer half-life time and higher bioavailability [1, 2]. For this reason, vitamin K2 is more effective in carboxylating extrahepatic vitamin K-dependent proteins than vitamin K1 [3]. Besides their role in the coagulation cascade, vitamin K-dependent proteins are involved in (patho)physiological calcification of the vasculature [4] and the bones [5, 6]. Matrix Gla Protein (MGP) is a vitamin K-dependent protein that inhibits vascular calcification and plays a regulatory role in the bones [7]. Osteocalcin is involved in bone metabolism [8]. Both MGP and osteocalcin require post-translational carboxylation to be fully functional and vitamin K is a co-factor for activation [7].

In the vasculature, calcifications are increasingly recognized as independent risk factors for cardiovascular disease. Current treatment of cardiovascular risk focuses on hypercholesterolemia, hypertension and thrombosis, but despite optimal treatment, the risk of new cardiovascular events remains high in patients with DM2 [9, 10]. Arterial calcifications can occur in both the intimal and medial layer of the vascular wall and these calcifications differ in cause and consequence. Intimal calcifications are generally considered as stabilized atherosclerotic plaques, whereas medial calcifications are thought to contribute to hypertension, arterial stiffness and cardiac hypertrophy [11]. Arterial calcifications, in any vascular bed, are associated with a three- to fourfold increase in the risk of cardiovascular events and progression of calcification is associated with more events [12, 13]. In addition, calcifications in different arterial beds have different clinical consequences. For example, calcification of the leg arteries is associated with peripheral arterial disease, intracranial calcifications are associated with stroke, and aortic calcifications are associated with arterial stiffness, stroke and decreased bone mineral density [14–16]. Reduction of arterial calcification progression in patients with cardiovascular diseases might, therefore, reduce the high residual cardiovascular risk. Several observational studies have shown an association of low vitamin K intake with vascular calcification and cardiovascular events, and MGP might form the link between these associations [17–19].

Osteocalcin is a marker for osteoblast activity and bone metabolism. MGP is also thought to be involved in bone metabolism, but its role is not fully understood. It is speculated to promote bone formation and inhibit osteoclast activity [20]. Despite inconsistent results on bone mineral density (BMD) [21, 22], vitamin K supplementation is shown to reduce clinical fractures [23] and this reduction might be caused by carboxylation and activation of osteocalcin and MGP [24].

Type 2 diabetes mellitus (DM2) is associated with increased arterial calcification and increased fractures [25, 26] and the low vitamin K status found in DM2 patients might contribute to this [27]. DM2 patients are prone to develop medial arterial calcifications. Since MGP is thought to inhibit the formation and progression of medial arterial calcifications, vitamin K supplementation might reduce calcification progression in these patients. Recently, we showed that 6 months vitamin K2 supplementation reduced circulating levels of inactive MGP, but did not halt arterial calcification as measured with ^18^F sodium-fluoride positron emission tomography (^18^F NaF PET) and computed tomography (CT) in the femoral arteries in patients with DM2 and a history of cardiovascular disease [28]. Different arterial beds, however, may be more or less prone to calcification and the role of MGP might differ between these different arteries [29]. This post hoc analysis aims to investigate the effect of 6 months vitamin K2 supplementation on systemic CT-measured arterial calcification and CT-measured BMD in the spine in patients with DM2 and cardiovascular disease.

## Methods

### Study design and population

This study is a pre-specified post hoc analysis of a double-blind, randomized, placebo-controlled clinical trial conducted at the University Medical Center Utrecht (UMCU) in the Netherlands. The methods have previously been published [28]. In short, patients were randomized to vitamin K2 or placebo. The primary end point of the original trial was change in active vascular calcification in the femoral artery as measured with ^18^F-NaF PET [28]. The sample size calculation was based on the primary outcome of the trial. A previous study into ^18^NaF PET/CT found a mean TBR of 1.96 [30]. Considering a power of 80%, a two-sided α of 5%, an SD of the difference in TBR between baseline and follow-up of 0.41 and a 15% dropout rate, 70 participants were required to detect a 15% difference in TBR. The outcomes of this post hoc analysis were change in arterial calcification mass scores and BMD as measured on conventional CT. Participants were recruited via the Diabetes Pearl String Initiative-cohort, a Julius Center database of patients interested in participating in studies and via the outpatient clinic of the UMCU and Diakonessenhuis Utrecht. Middle-aged men and women (> 40), diagnosed with DM2 and with known pre-existing arterial disease, were included. Pre-existing arterial disease was based on an ankle-brachial index (ABI) < 0.9 and/or was diagnosed by a physician. Exclusion criteria were vitamin K antagonist use, the use of vitamin K containing (multi)vitamins and unwillingness to stop before randomization, coagulation problems (e.g., deep venous thrombosis) and an estimated glomerular filtration rate (eGFR) below 30 ml/kg/min. Randomization was performed stratified by sex by the data management department. The code was kept at the data management and the pharmacy departments. The study was approved by the medical ethical review board of the UMC Utrecht and registered in clinical trial registries (NCT02839044/NTR5287). All participants gave written informed consent.

### Intervention

Participants were randomized in a 1:1 ratio to 360 µg/day vitamin K or placebo treatment as previously described [28]. The vitamin K tablets contained 360 µg menaquinone-7, the placebo tablets contained the same raw material and were similar in taste and appearance. Menaquinone-7 (Vitamin K2) was used because of its higher bioavailability and longer half-life time compared to phylloquinones (vitamin K1) [3]. The dose used in this study is at the high end of what can be achieved through diet and has previously been shown to effectively decrease circulating inactive dephosphorylated-uncarboxylated (dp-uc)MGP levels without affecting coagulation [31]. Leftover vitamin K and placebo tablets were returned after the trial and the compliance was estimated as the number of tablets that were returned divided by the number of tablets that should have been taken × 100. The compliance was high in both the vitamin K group (*β* [95% CI]: 97.4% [92.3%; 99.1%] and placebo arm (*β* [95% CI]: 97.8% [94.2%; 99.7%] [28].

### Dp-ucMGP level determination

Serum dp-ucMGP levels were determined with a sandwich ELISA using the IDS Automated Analyser IDS-iSYS InaKtif MGP assay (Maastricht University, The Netherlands).

### Computed tomography (CT) acquisition and analysis

All participants underwent a low-dose, full-body CT scan at baseline and 6-month follow-up (Siemens, Healthcare, Erlangen, Germany). Arterial calcification mass was quantified in several larger arterial beds that can reliably be quantified on low-dose CT scans. The arteries were the intracranial and common carotid arteries, the coronary arteries, the aorta, the iliac arteries and the arteries of the legs (femoral and crural arteries combined). The findings in the femoral arteries were the primary outcome of the trial and were published elsewhere [28]. The calcification mass score was measured using an in-house developed program (iX Viewer, Image Science Institute, Utrecht, The Netherlands) as previously described [32, 33] The observer manually identified potential calcifications that were segmented using a threshold of 130 Hounsfield Units (HU) for calcification. The calcification mass score was computed as the product of the volume of the lesion (in ml) and the mean attenuation (in HU) of the lesion. Bone mineral density was assessed using an in-house developed software tool as described previously [34]. Thoracic and lumbar vertebrae were automatically segmented using an iterative segmentation approach. Fractures were visually identified and defined as > 20% height loss of one the pillars of the vertebral body. BMD of the trabecular core of the non-fractured vertebra was expressed as the mean HU in all thoracic and lumbar vertebrae.

### Statistical analysis

Descriptive data are presented as categorical variables (*n*, %), normally distributed continuous (mean ± SD) or non-normally distributed (median, interquartile range). The difference in absolute and relative change in CT-based calcification mass score or BMD between the treatment and placebo group was analyzed with the Mann–Whitney *U* test. The relative change was calculated with the following formula: (*x* follow-up − *x* baseline)/*x* baseline × 100. In addition, to adjust for baseline differences, calcification mass was log + 1-transformed and linear regression models were built. These models included treatment (vitamin K or placebo, vitamin K as the reference) and log-transformed calcification mass of the individual arteries or BMD at baseline as the independent variables and log-transformed calcification mass or BMD after 6 months as the dependent variable (e.g., arterial calcification mass FU ~ treatment (vitamin K or placebo) + arterial calcification mass baseline). A *p* value < 0.05 was regarded statistically significant. All analyses were performed in SPSS version 25.0, figures were made in Rstudio v1.1.456.

## Results

### Trial population

Sixty-eight eligible patients were randomized: 35 to the intervention and 33 to the placebo arm. Mean age was 69 years in both groups; 74% vs 79% were male in the intervention and the placebo groups, respectively. In the intervention group, two patients were lost to follow-up versus six patients in the placebo group. The loss to follow-up was unrelated to the intervention and was mostly due to discomfort with the PET/CT scan. Segmentation failed in 98 of the 2176 vertebrae (5%) and 14 (0.6%) of the segmented vertebrae were fractured and therefore excluded. Baseline characteristics of the intervention and placebo group are presented in Table 1.

**Table 1:** Baseline characteristics of the trial population

Characteristic	Vitamin K (*n* = 35)	Placebo (*n* = 33)
Age, years	69 ± 8	69 ± 8
Male sex, *n* (%)	26 (74)	26 (79)
Body mass index	31 ± 6	31 ± 5
Systolic blood pressure, mmHg	136 ± 21	138 ± 14
Diastolic blood pressure, mmHg	70 ± 11	74 ± 9
Current smokers, *n* (%)	6 (17)	4 (12)
Previous smokers, *n* (%)	24 (67)	18 (55)
Never smokers, *n* (%)	5 (15)	11 (33)
Medical history		
Cerebrovascular disease, *n* (%)	12 (34)	11 (33)
Coronary artery disease, *n* (%)	23 (66)	18 (55)
Peripheral arterial disease, *n* (%)	6 (17)	10 (30)
Abdominal aortic aneurysm, *n* (%)	2 (6)	4 (12)
Medication		
Antihypertensive, *n* (%)	30 (86)	30 (90)
Glucose lowering, *n* (%)	30 (86)	30 (90)
Lipid lowering, *n* (%)	16 (46)	15 (46)
Laboratory measurements		
HbA1c, mmol/mol	57 ± 15	60 ± 17
Total cholesterol, mmol/l	4.5 ± 1.3	4.2 ± 1.2
LDL cholesterol, mmol/l	2.1 ± 0.9	2.0 ± 0.9
HDL cholesterol, mmol/l	1.1 ± 0.3	1.1 ± 0.3
Triglycerides, mmol/l	2.8 (1.8–3.4)	1.9 (1.5–2.7)
dp-ucMGP, pmol/l	613 (513–684)	615 (489–743)
Arterial calcification		
Intracranial internal carotid artery, mass score	12 (3–25)	3 (0–35)
Common carotid artery, mass score	3 (0–25)	2 (0–10)
Coronary arteries, mass score	74 (13–165)	46 (1–148)
Aorta, mass score	742 (322–1337)	365 (39–1144)
Iliac arteries, mass score	633 (242–1148)	337 (66–764)
Leg arteries, mass score	309 (93–851)	90 (11–627)
Total arterial calcification, mass score	1694 (812–3584)	1182 (235–2445)
Bone mineral density		
Thoracic vertebrae, HU	162 ± 44	166 ± 45
Lumbar vertebrae, HU	126 ± 36	133 ± 48
All vertebrae, HU	151 ± 39	156 ± 43

Values are *n* (%), mean ± SD or median (interquartile range)

*HDL* high density lipoprotein, *LDL* low density lipoprotein, *dp-ucMGP* = dephosphorylated uncarboxylated matrix glycoprotein, *HU* Hounsfield units

Despite randomization, participants in the vitamin K arm had higher arterial calcification mass scores in all arterial beds compared to the placebo arm at baseline. These differences were statistically significant in the aorta [median (IQR): 742 (322–1337) vs 365 (39–1144), *p* = 0.05], iliac arteries [median (IQR): 633 (242–1148) vs 337 (66–764), *p* = 0.02] and the total arterial calcification mass score [median (IQR): 1694 (812–3584) vs 1182 (235–2445), *p* = 0.03] and was nearly significant in the calcification mass score of the legs [median (IQR): 309 (93–851) vs 90 (11–627), *p* = 0.07].

At baseline, no difference in plasma dp-ucMGP [613 (513–684) pmol/l vs. 615 (489–743) pmol/l, *p* = 0.96] and BMD (all vertebrae: 151 ± 39 HU vs. 156 ± 43 HU, *p* = 0.62) was found between the vitamin K and placebo arm, respectively (Table 1).

### The effect of vitamin K supplementation on arterial calcification mass and BMD

No significant difference in arterial calcification progression between the vitamin K arm and the placebo arm was found for any arterial bed, although a trend toward lower progression in the placebo arm was found in the iliac arteries [median [IQR): 25 (6; 87) mg vs 5 (− 4; 30) mg, *p* = 0.07]. In addition, no difference in BMD decline was found between the groups [median (IQR): 3 (− 2; 16) HU vs − 1 (− 5; 10) HU, *p* = 0.24] in the vitamin K and placebo arm, respectively (Table 2).

**Table 2:** Difference in arterial calcification mass score and bone mineral density after 6 months of vitamin K treatment or placebo

Arterial calcification mass	Difference baseline and follow-up			Linear regression models adjusted for baseline measurements
	Vitamin K, *N* = 33	Placebo, *N* = 27	*p*	*β* [95% CI]
Intracranial internal carotid artery	0 [− 1; 5)	0 (− 0; 2)	0.76	− 0.03 [− 0.21; 0.15]
Common carotid artery	0 (− 0; 3)	0 (− 1; 0)	0.20	− 0.14 [− 0.48; 0.21]
Coronary arteries	5 (− 5; 12)	1 (− 4; 11)	0.68	− 0.01 [− 0.20; 0.17]
Aorta	40 (− 30; 125)	11 (0; 47)	0.55	0.02 [− 0.06; 0.11]
Iliac arteries	25 (6; 87)	5 (− 4; 30)	0.07	− 0.02 [− 0.08; 0.05]
Leg arteries	35 (− 8; 99)	7 (0; 47)	0.62	− 0.03 [− 0.21; 0.14]
Total arterial calcification score	84 (− 37; 206)	36 (1; 129)	0.38	− 0.02 [− 0.10; 0.06]
Bone mineral density				
Thoracic	5 (− 3; 15)	− 2 (− 5; 12)	0.28	− 1.95 [− 11.65; 7.75]
Lumbar	3 (− 5; 18)	1 (− 5; 6)	0.55	− 2.40 [− 11.26; 6.64]
Total BMD	3 (− 2; 16)	− 1 (− 5; 10)	0.24	− 2.06 [− 11.26; 7.30]

Data ares presented as median (IQR) for non-normally distributed variables. The difference in arterial calcification mass score progression and BMD decline between the vitamin K and placebo groups were estimated with the Mann–Whitney *U* test. In addition, to adjust for baseline differences, linear regression models were built with vitamin K and placebo treatment and log-transformed calcification mass or BMD at baseline as the determinants and log-transformed calcification mass or BMD after 6 months of follow-up as the outcome

**Fig. 1:**
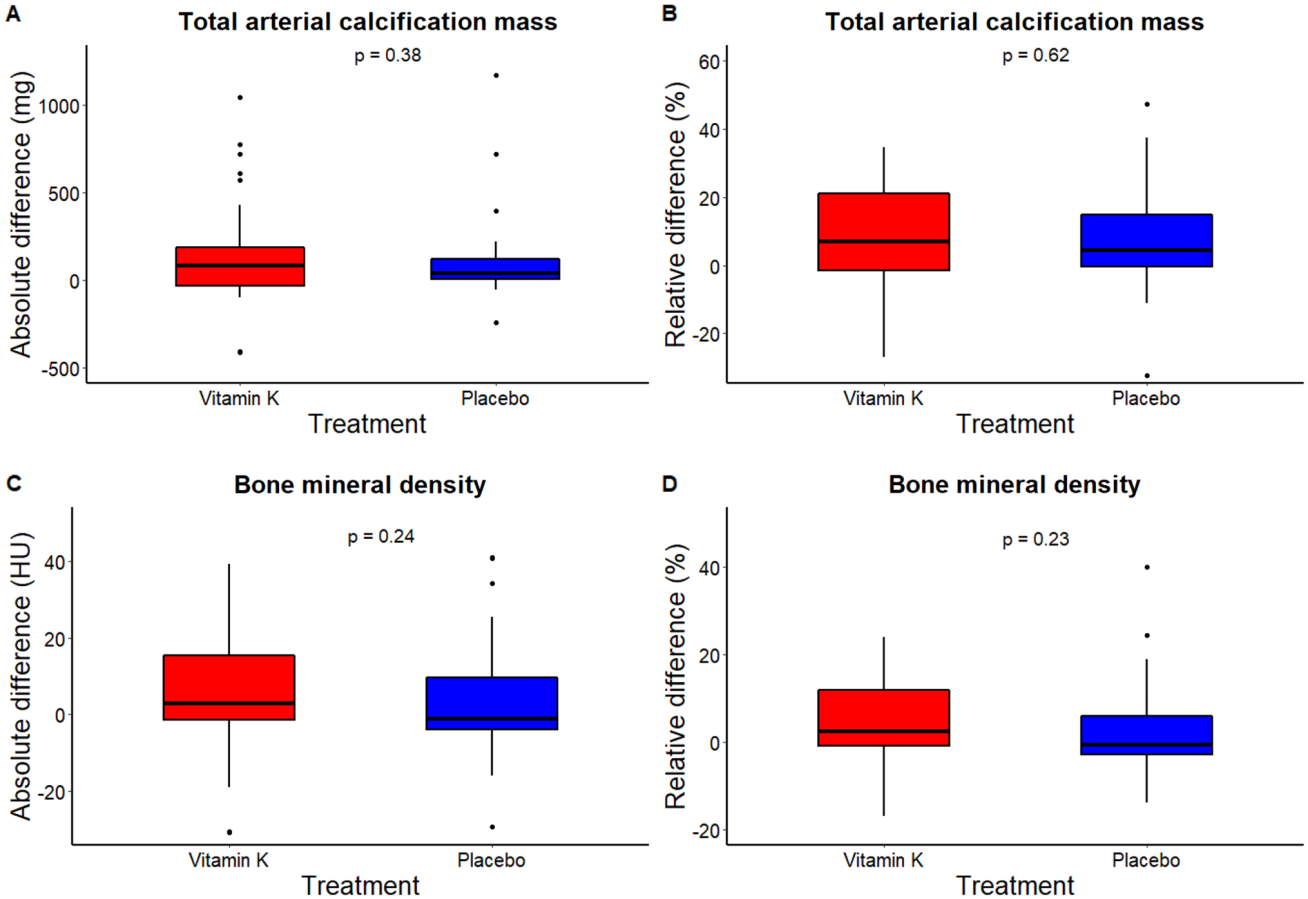
No difference was found in absolute (**a**, **c**) and relative (**b**, **d**) differences in total body arterial calcification mass (**a**, **b**) and bone mineral density (**c**, **d**) after 6 months of vitamin K treatment (red) or placebo (blue)

When adjusted for baseline arterial calcification mass scores, 6 months of vitamin K treatment did not halt progression of arterial calcification in any arterial bed when compared to the placebo (*β*: − 0.02; 95% CI: − 0.10; 0.06 mg; *p* = 0.64 for the total arterial calcification mass). No effect of vitamin K treatment on BMD was found (*β*: − 2.06; 95% CI: − 11.26; 7.30 HU; *p* = 0.66 for all vertebrae). See Fig. 1 and Table 2 for individual arterial beds and vertebral and lumbar vertebrae.

## Discussion

This study shows that 6 months of vitamin K supplementation does not affect CT-measured arterial calcification or CT-measured BMD in patients with DM2 and a history of cardiovascular disease. Although vitamin K supplementation reduced circulating inactive MGP, as was shown in the original trial, our findings do not support that vitamin K supplementation has a role in the prevention of arterial calcification progression or osteoporosis in patients with DM2, but some limitations have to be acknowledged.

Several clinical trials investigating the effect of vitamin K supplementation on arterial calcification have been performed. Despite a consistent decrease in the circulating inactive form of MPG (dephosphorylated-uncarboxylated (dp-uc) MGP) after vitamin K supplementation [28, 31, 35, 36], these trials showed differing results on arterial calcification progression. One year treatment with vitamin K2 in hemodialysis patients did not affect aortic calcification progression [37], although the dose in this study was lower compared to our study. Another study with 3-year follow-up did not find an effect of multivitamin treatment with vitamin K1 when compared to multivitamin alone on the progression of coronary artery calcification, although a subgroup analysis showed a small effect in patients that were > 85% adherent to the treatment [38]. A meta-analysis of clinical trials investigating the effect of vitamin K on calcification progression showed a positive effect of vitamin K treatment on arterial calcification progression [39]. This effect was mainly driven by a study into the effect of vitamin K on aortic valve calcification which showed that 12 months treatment with 2 mg vitamin K1 reduced the progression of aortic valve calcification (10% progression in vitamin K1 vs 22% in the placebo group) in 72 patients with aortic valve stenosis [40].

In our trial, there was a significant difference in arterial calcification mass scores in several arterial beds between the intervention and placebo groups despite randomization. Although we adjusted for these differences in the regression models, this might have affected the outcomes since—from the coronary arteries—it is known that baseline calcification mass is a strong predictor of calcification progression [41]. Another explanation might be that our patients did not have very severe vitamin K deficiency. Although serum dp-ucMGP levels in this population were relatively high when compared to the healthy population of comparable age (372–376 pmol/l) [27, 42], much higher levels up to 1100 pmol/l are reported in patients with chronic kidney disease [43, 44]. Selection of patients with severe vitamin K deficiency might be warranted for future trials.

Despite these differing results on calcification progression, several trials showed a reduction of arterial stiffness progression with vitamin K supplementation [36, 39, 45]. This challenges our understanding of the role of MGP in vascular physiology. Since calcification is shown to add to arterial stiffness, the positive effects in these trials are thought to be mediated by a reduction in arterial calcification [46]. Possibly, MGP is involved in other mechanisms that contribute to arterial stiffness, including reduced elastin degradation and collagen formation in the arterial wall, as well [47].

We could not confirm a positive effect of vitamin K supplementation on BMD in patients with type 2 diabetes, although the duration of treatment was relatively short and we used CT scans instead of DXA scans for the measurements. Some clinical trials have shown an increase of BMD after vitamin K treatment [21, 48], whereas others did not [22, 49]. A meta-analysis of 36 clinical trials that investigated the effect of vitamin K treatment on bone mineral density and fractures showed vitamin K treatment does not affect BMD in postmenopausal women, although it may reduce clinical fractures [23]. This suggests that vitamin K plays a role in bone architecture and quality, rather than in BMD. DM2 patients have an increased risk of fractures despite normal or even increased BMD [50]. Future studies might therefore include the effect of vitamin K on bone structure and fractures, rather than on BMD [51].

The strengths of this study include the randomized controlled design, excellent compliance and extensive measurements of systemic arterial calcification and that we used CT scans to quantify vertebral BMD. Although areal BMD (aBMD) as measured with DXA is currently the golden standard for the measurement of BMD, and CT scans provide the opportunity to exclude the cortical bone from the analysis. Both low aBMD as measured with DXA and volumetric BMD as measured with CT are shown to be associated with increased fractures, but vBMD of the lumbar spine has been shown to be a better predictor for spine fractures than areal BMD as measured with DXA scans [52].

However, several limitations should be acknowledged. The relatively high dropout rate, especially in the placebo arm, might have affected the statistical power. Since the sample size calculation of this study was based on active arterial calcification progression, as measured with ^18^NaF PET/CT, it might have been underpowered to detect changes in BMD and CT-measured calcifications in this relatively short follow-up period. The reasons for dropout were mainly due to discomfort with the ^18^NaF PET/CT. Future clinical trials might want to consider using conventional CT instead of ^18^NaF PET/CT, since this requires shorter scan time, no radiotracer needs to be infused and it has a lower radiation dose. Since the baseline characteristics of the participants that fulfilled the follow-up period were similar to the baseline characteristics of all participants that were randomized, we do not think that selection bias has affected our results. Since we included DM2 patients with cardiovascular diseases, and approximately 75% of our participants were male, our results might not be generalizable to broader populations. Since we quantified arterial calcification mass on whole body CT scans without ECG-gated cardiac CT, the coronary artery calcification mass scores might not be accurate and future research into the effect of vitamin K on coronary artery calcification progression should consider using triggered scans or more advanced motion and partial volume correction [53, 54].

In conclusion, 6 months of vitamin K supplementation did not halt progression of arterial calcification and did not affect BMD in patients with DM2 and cardiovascular disease. Given the limitations of our study future clinical trials may want to pre-select patients with very low vitamin K status and longer follow-up time might be warranted.

## References

[1] Halder M, Petsophonsakul P, Akbulut AC, Pavlic A, Bohan F, Anderson E, Maresz K, Kramann R, Schurgers L (2019). Vitamin K: double bonds beyond coagulation insights into differences between vitamin K1 and K2 in health and disease. Int J Mol Sci.

[2] Schurgers LJ, Vermeer C (2000). Determination of phylloquinone and menaquinones in food. Effect of food matrix on circulating vitamin K concentrations. Haemostasis.

[3] Schurgers LJ, Teunissen KJ, Hamulyak K, Knapen MH, Vik H, Vermeer C (2007). Vitamin K-containing dietary supplements: comparison of synthetic vitamin K1 and natto-derived menaquinone-7. Blood.

[4] Vossen LM, Kroon AA, Schurgers LJ, de Leeuw PW (2019). Pharmacological and nutritional modulation of vascular calcification. Nutrients.

[5] Myneni VD, Mezey E (2017). Regulation of bone remodeling by vitamin K2. Oral Dis.

[6] Cozzolino M, Fusaro M, Ciceri P, Gasperoni L, Cianciolo G (2019). The role of vitamin K in vascular calcification. Adv Chronic Kidney Dis.

[7] Schurgers LJ, Uitto J, Reutelingsperger CP (2013). Vitamin K-dependent carboxylation of matrix Gla-protein: a crucial switch to control ectopic mineralization. Trends Mol Med.

[8] Zoch ML, Clemens TL, Riddle RC (2016). New insights into the biology of osteocalcin. Bone.

[9] Kaasenbrood L, Boekholdt SM, van der Graaf Y, Ray KK, Peters RJ, Kastelein JJ, Amarenco P, LaRosa JC, Cramer MJ, Westerink J, Kappelle LJ, de Borst GJ, Visseren FL (2016). Distribution of estimated 10-year risk of recurrent vascular events and residual risk in a secondary prevention population. Circulation.

[10] Mazzone T, Chait A, Plutzky J (2008). Cardiovascular disease risk in type 2 diabetes mellitus: insights from mechanistic studies. Lancet.

[11] Lanzer P, Boehm M, Sorribas V, Thiriet M, Janzen J, Zeller T, St Hilaire C, Shanahan C (2014). Medial vascular calcification revisited: review and perspectives. Eur Heart J.

[12] Kramer CK, Zinman B, Gross JL, Canani LH, Rodrigues TC, Azevedo MJ, Retnakaran R (2013). Coronary artery calcium score prediction of all cause mortality and cardiovascular events in people with type 2 diabetes: systematic review and meta-analysis. BMJ.

[13] Rennenberg RJ, Kessels AG, Schurgers LJ, van Engelshoven JM, de Leeuw PW, Kroon AA (2009). Vascular calcifications as a marker of increased cardiovascular risk: a meta-analysis. Vasc Health Risk Manag.

[14] Ho CY, Shanahan CM (2016). Medial arterial calcification: an overlooked player in peripheral arterial disease. Arterioscler Thromb Vasc Biol.

[15] Bos D, Portegies ML, van der Lugt A, Bos MJ, Koudstaal PJ, Hofman A, Krestin GP, Franco OH, Vernooij MW, Ikram MA (2014). Intracranial carotid artery atherosclerosis and the risk of stroke in whites: the Rotterdam Study. JAMA Neurol.

[16] Bartstra JW, Mali WP, Spiering W, de Jong PA (2020). Abdominal aortic calcification: from ancient friend to modern foe. Eur J Prev Cardiol.

[17] Dalmeijer GW, van der Schouw YT, Magdeleyns EJ, Vermeer C, Verschuren WM, Boer JM, Beulens JW (2013). Matrix Gla protein species and risk of cardiovascular events in type 2 diabetic patients. Diabetes Care.

[18] Pivin E, Ponte B, Pruijm M, Ackermann D, Guessous I, Ehret G, Liu YP, Drummen NE, Knapen MH, Pechere-Bertschi A, Paccaud F, Mohaupt M, Vermeer C, Staessen JA, Vogt B, Martin PY, Burnier M, Bochud M (2015). Inactive matrix Gla-protein is associated with arterial stiffness in an adult population-based study. Hypertension.

[19] Chen HG, Sheng LT, Zhang YB, Cao AL, Lai YW, Kunutsor SK, Jiang L, Pan A (2019). Association of vitamin K with cardiovascular events and all-cause mortality: a systematic review and meta-analysis. Eur J Nutr.

[20] Zhang J, Ma Z, Yan K, Wang Y, Yang Y, Wu X (2019). Matrix Gla protein promotes the bone formation by up-regulating Wnt/beta-catenin signaling pathway. Front Endocrinol (Lausanne).

[21] Knapen MH, Drummen NE, Smit E, Vermeer C, Theuwissen E (2013). Three-year low-dose menaquinone-7 supplementation helps decrease bone loss in healthy postmenopausal women. Osteoporos Int.

[22] Booth SL, Dallal G, Shea MK, Gundberg C, Peterson JW, Dawson-Hughes B (2008). Effect of vitamin K supplementation on bone loss in elderly men and women. J Clin Endocrinol Metab.

[23] Mott A, Bradley T, Wright K, Cockayne ES, Shearer MJ, Adamson J, Lanham-New SA, Torgerson DJ (2019). Effect of vitamin K on bone mineral density and fractures in adults: an updated systematic review and meta-analysis of randomised controlled trials. Osteoporos Int.

[24] Rodriguez-Olleros Rodriguez C, Diaz Curiel M (2019). Vitamin K and bone health: a review on the effects of vitamin K deficiency and supplementation and the effect of non-vitamin K antagonist oral anticoagulants on different bone parameters. J Osteoporos.

[25] Napoli N, Chandran M, Pierroz DD, Abrahamsen B, Schwartz AV, Ferrari SL, Bone IOF, Diabetes Working G (2017). Mechanisms of diabetes mellitus-induced bone fragility. Nat Rev Endocrinol.

[26] Yahagi K, Kolodgie FD, Lutter C, Mori H, Romero ME, Finn AV, Virmani R (2017). Pathology of human coronary and carotid artery atherosclerosis and vascular calcification in diabetes mellitus. Arterioscler Thromb Vasc Biol.

[27] Riphagen IJ, Keyzer CA, Drummen NEA, de Borst MH, Beulens JWJ, Gansevoort RT, Geleijnse JM, Muskiet FAJ, Navis G, Visser ST, Vermeer C, Kema IP, Bakker SJL (2017). Prevalence and effects of functional vitamin K insufficiency: the PREVEND study. Nutrients.

[28] Zwakenberg SR, de Jong PA, Bartstra JW, van Asperen R, Westerink J, de Valk H, Slart R, Luurtsema G, Wolterink JM, de Borst GJ, van Herwaarden JA, van de Ree MA, Schurgers LJ, van der Schouw YT, Beulens JWJ (2019). The effect of menaquinone-7 supplementation on vascular calcification in patients with diabetes: a randomized, double-blind, placebo-controlled trial. Am J Clin Nutr.

[29] Barrett H, Okeeffe M, Kavanagh E, Walsh M, Oconnor EM (2018). Is matrix Gla protein associated with vascular calcification? A systematic review. Nutrients.

[30] Janssen T, Bannas P, Herrmann J, Veldhoen S, Busch JD, Treszl A, Munster S, Mester J, Derlin T (2013). Association of linear (1)(8)F-sodium fluoride accumulation in femoral arteries as a measure of diffuse calcification with cardiovascular risk factors: a PET/CT study. J Nucl Cardiol.

[31] Dalmeijer GW, van der Schouw YT, Magdeleyns E, Ahmed N, Vermeer C, Beulens JW (2012). The effect of menaquinone-7 supplementation on circulating species of matrix Gla protein. Atherosclerosis.

[32] Rutten A, Isgum I, Prokop M (2008). Coronary calcification: effect of small variation of scan starting position on Agatston, volume, and mass scores. Radiology.

[33] Bartstra JW, de Jong PA, Kranenburg G, Wolterink JM, Isgum I, Wijsman A, Wolf B, den Harder AM, Mali W, Spiering W (2020). Etidronate halts systemic arterial calcification in pseudoxanthoma elasticum. Atherosclerosis.

[34] Lessmann N, van Ginneken B, de Jong PA, Isgum I (2019). Iterative fully convolutional neural networks for automatic vertebra segmentation and identification. Med Image Anal.

[35] Mansour AG, Hariri E, Daaboul Y, Korjian S, El Alam A, Protogerou AD, Kilany H, Karam A, Stephan A, Bahous SA (2017). Vitamin K2 supplementation and arterial stiffness among renal transplant recipients-a single-arm, single-center clinical trial. J Am Soc Hypertens.

[36] Knapen MH, Braam LA, Drummen NE, Bekers O, Hoeks AP, Vermeer C (2015). Menaquinone-7 supplementation improves arterial stiffness in healthy postmenopausal women. A double-blind randomised clinical trial. Thromb Haemost.

[37] Oikonomaki T, Papasotiriou M, Ntrinias T, Kalogeropoulou C, Zabakis P, Kalavrizioti D, Papadakis I, Goumenos DS, Papachristou E (2019). The effect of vitamin K2 supplementation on vascular calcification in haemodialysis patients: a 1-year follow-up randomized trial. Int Urol Nephrol.

[38] Shea MK, O'Donnell CJ, Hoffmann U, Dallal GE, Dawson-Hughes B, Ordovas JM, Price PA, Williamson MK, Booth SL (2009). Vitamin K supplementation and progression of coronary artery calcium in older men and women. Am J Clin Nutr.

[39] Lees JS, Chapman FA, Witham MD, Jardine AG, Mark PB (2019). Vitamin K status, supplementation and vascular disease: a systematic review and meta-analysis. Heart.

[40] Brandenburg VM, Reinartz S, Kaesler N, Kruger T, Dirrichs T, Kramann R, Peeters F, Floege J, Keszei A, Marx N, Schurgers LJ, Koos R (2017). Slower progress of aortic valve calcification with vitamin K supplementation: results from a prospective interventional proof-of-concept study. Circulation.

[41] Diederichsen SZ, Gronhoj MH, Mickley H, Gerke O, Steffensen FH, Lambrechtsen J, Ronnow Sand NP, Rasmussen LM, Olsen MH, Diederichsen A (2017). CT-detected growth of coronary artery calcification in asymptomatic middle-aged subjects and association with 15 biomarkers. JACC Cardiovasc Imaging.

[42] Machado-Fragua MD, Hoogendijk EO, Struijk EA, Rodriguez-Artalejo F, Lopez-Garcia E, Beulens JW, van Ballegooijen AJ (2020). High dephospho-uncarboxylated matrix Gla protein concentrations, a plasma biomarker of vitamin K, in relation to frailty: the longitudinal aging study Amsterdam. Eur J Nutr.

[43] Schurgers LJ, Barreto DV, Barreto FC, Liabeuf S, Renard C, Magdeleyns EJ, Vermeer C, Choukroun G, Massy ZA (2010). The circulating inactive form of matrix gla protein is a surrogate marker for vascular calcification in chronic kidney disease: a preliminary report. Clin J Am Soc Nephrol.

[44] Kurnatowska I, Grzelak P, Masajtis-Zagajewska A, Kaczmarska M, Stefanczyk L, Vermeer C, Maresz K, Nowicki M (2015). Effect of vitamin K2 on progression of atherosclerosis and vascular calcification in nondialyzed patients with chronic kidney disease stages 3–5. Pol Arch Med Wewn.

[45] Braam LA, Hoeks AP, Brouns F, Hamulyak K, Gerichhausen MJ, Vermeer C (2004). Beneficial effects of vitamins D and K on the elastic properties of the vessel wall in postmenopausal women: a follow-up study. Thromb Haemost.

[46] Tsao CW, Pencina KM, Massaro JM, Benjamin EJ, Levy D, Vasan RS, Hoffmann U, O'Donnell CJ, Mitchell GF (2014). Cross-sectional relations of arterial stiffness, pressure pulsatility, wave reflection, and arterial calcification. Arterioscler Thromb Vasc Biol.

[47] Cocciolone AJ, Hawes JZ, Staiculescu MC, Johnson EO, Murshed M, Wagenseil JE (2018). Elastin, arterial mechanics, and cardiovascular disease. Am J Physiol Heart Circ Physiol.

[48] Je SH, Joo NS, Choi BH, Kim KM, Kim BT, Park SB, Cho DY, Kim KN, Lee DJ (2011). Vitamin K supplement along with vitamin D and calcium reduced serum concentration of undercarboxylated osteocalcin while increasing bone mineral density in Korean postmenopausal women over sixty-years-old. J Korean Med Sci.

[49] Volpe SL, Leung MM, Giordano H (2008). Vitamin K supplementation does not significantly impact bone mineral density and biochemical markers of bone in pre- and perimenopausal women. Nutr Res.

[50] Iwamoto J, Sato Y, Takeda T, Matsumoto H (2011). Bone quality and vitamin K2 in type 2 diabetes: review of preclinical and clinical studies. Nutr Rev.

[51] Chang G, Boone S, Martel D, Rajapakse CS, Hallyburton RS, Valko M, Honig S, Regatte RR (2017). MRI assessment of bone structure and microarchitecture. J Magn Reson Imaging.

[52] Chalhoub D, Orwoll ES, Cawthon PM, Ensrud KE, Boudreau R, Greenspan S, Newman AB, Zmuda J, Bauer D, Cummings S, Cauley JA, Osteoporotic Fractures in Men Study Research G (2016). Areal and volumetric bone mineral density and risk of multiple types of fracture in older men. Bone.

[53] Sprem J, de Vos BD, Lessmann N, de Jong PA, Viergever MA, Isgum I (2018). Impact of automatically detected motion artifacts on coronary calcium scoring in chest computed tomography. J Med Imaging (Bellingham).

[54] Sprem J, de Vos BD, Lessmann N, van Hamersvelt RW, Greuter MJW, de Jong PA, Leiner T, Viergever MA, Isgum I (2018). Coronary calcium scoring with partial volume correction in anthropomorphic thorax phantom and screening chest CT images. PLoS ONE.

